# Editorial: Impact of acoustic environments and noise on auditory perception

**DOI:** 10.3389/fnins.2026.1822307

**Published:** 2026-03-23

**Authors:** Mary M. Flaherty, Achim Klug

**Affiliations:** 1 University of Illinois at Urbana-Champaign, Champaign, IL, United States; 2 Department of Physiology & Biophysics, University of Colorado Anschutz Medical Campus, Aurora, CO, United States

**Keywords:** acoustic environment, auditory masking, auditory perception, cocktail party, speech in noise

The 16 articles assembled in this Research Topic share a common tension. The auditory system is, by any measure, a remarkable piece of biological engineering—capable of parsing speech, localizing sources, and tracking melody across extraordinary ranges of acoustic complexity. And yet it is also surprisingly vulnerable: to noise, to time, to the particular acoustic pressures of the world we now inhabit. Spanning peripheral physiology, neural processing, psychoacoustics, cognitive neuroscience, and public health, the contributions here resist any single framing. What they offer instead is a multi-level portrait that moves between molecular mechanisms and ecological context, between the controlled laboratory and the noisy real world.

Behavioral studies in this Research Topic show clearly that acoustic environments determine which perceptual cues listeners can actually use. Spatial separation, voice characteristics, suprasegmental prosodic structure, presentation level—none of these operate in isolation, and none reliably predicts outcomes across all conditions. Cues that help in one environment may be unavailable or ineffective in another. More importantly, improvements in recognition accuracy do not always reflect reduced processing demands. These findings suggest that noisy and complex acoustic environments alter not only what listeners perceive, but how perceptual and cognitive resources are engaged.

This dissociation between what listeners get right and what it costs them to do so runs through many of the papers here. Measures of listening effort frequently remain elevated even when recognition performance improves, a finding that should give pause to anyone who uses accuracy as a proxy for processing demand. Neural measures sharpen the picture further. Spectrally degraded speech disrupts later cortical processes tied to semantic integration and decision-making. Neural encoding of speech sounds can deteriorate under noise and aging without any corresponding decline in perceptual discrimination scores. The auditory system, in other words, can compensate, but compensation is not free, and behavioral outcomes alone do not reveal what it costs or where it breaks down.

Several contributions go beyond immediate perceptual effects to ask what happens over longer timescales. Chronic noise exposure reshapes the auditory system in ways that are not fully reversible. Across epidemiological, physiological, and neuroimaging approaches, sustained noise is shown to increase vulnerability to hearing damage, alter brain structure, and affect long-term hearing health even in populations for whom noise is simply part of daily life. Acoustic environments are not only perceptual challenges. They are cumulative biological stressors, and their effects accumulate accordingly.

Nowhere is this clearer than in Coco et al.'s scoping review of noise-induced hearing loss (NIHL) among farmworkers. Across 57 studies spanning six decades, prevalence rates as range from 46 to 98%, a spread so wide it is itself a finding. That range reflects measurement heterogeneity that has prevented meaningful cross-study comparisons for decade, a structural problem the WHO has been working to address since 1991 and one that remains unresolved. That figures this high have not improved despite growing awareness, is in itself, a finding worth sitting with.

Two papers approach biological noise vulnerability from different directions. Song et al. review the evidence for a cochlear circadian clock, making the case that susceptibility to acoustic trauma varies across the day. Nighttime noise exposure triggers permanent threshold shifts where equivalent daytime exposure does not—a difference tied to oscillations in BDNF and glucocorticoid signaling. The practical implications are still being worked out, but the finding complicates any assumption that time of day is irrelevant to noise exposure guidelines. Murray et al. take a more ecological approach, documenting temporary threshold shifts following concert attendance. Post-event tinnitus was substantial. The risks of recreational noise exposure are not new, but real-world quantification of this kind remains surprisingly rare. Wang et al. extend these consequences to the brain itself, showing enhanced small-world structural network properties in NIHL patients—changes that may reflect compensatory remodeling—and identifying correlations between network topology and inflammatory and coagulation markers. The picture that emerges is of peripheral cochlear damage feeding into systemic inflammation and, ultimately, central reorganization with potential consequences for cognitive health well beyond hearing.

Two studies from the University of Oldenburg offer the most mechanistically detailed account of how aging affects speech encoding across levels of the auditory system. Heeringa et al. report something that initially seems wrong: auditory nerve fibers in quiet-aged gerbils show enhanced temporal coding of vowels. The resolution is elegant. Elevated thresholds place fixed-level stimuli closer to the optimal operating point for phase-locking, so peripheral encoding actually improves. And yet behavioral vowel discrimination stays the same. The bottleneck, they argue, is central. Jüchter et al. extend this work across species. Gerbils and humans share strikingly similar vowel perceptual maps organized along formant dimensions, but age-related consonant discrimination deficits appear only in humans. That asymmetry is likely not coincidental: laboratory-raised gerbils do not accumulate decades of noise exposure, and their central auditory systems age differently as a result. These two papers together push back against the common assumption that age-related speech-in-noise difficulty originates primarily in the cochlea. It is a more mixed story—peripheral encoding changes interacting with central constraints, especially for the temporally demanding consonant features that matter most for intelligibility.

Several other contributions take on degraded speech from distinct angles but converge on a shared finding: that cue utilization, cognitive load, and listener characteristics interact in ways that single-measure paradigms reliably miss. Oh et al. show that presentation level modulates the relative weighting of voice-gender and spatial cues, with cue utilization shifting alongside signal characteristics in ways that matter for hearing aid and cochlear implant design. Choi et al. demonstrate that spectral degradation reaches beyond acoustics to affect late-stage cortical processing, with N2 and P3b amplitudes scaling reliably with spectral channel count. These components may offer clinically useful physiological markers of cognitive load under degraded conditions. Kwon and Yang bring this into the classroom, documenting how face masks affected speech recognition in 6-year-old children in ways that depended on talker gender, listener gender, mask type, and room acoustics. KF94 masks eliminated the female-talker advantage that girls otherwise show. The interaction between signal degradation and listener characteristics during language development is not a laboratory abstraction. Shen and DeDe take a methodological step forward by combining offline accuracy with online pupillometry to study how suprasegmental and lexico-semantic cues jointly facilitate speech recognition under informational masking. Their key finding: the “cue reliance” and “resource limitation” hypotheses are not competing explanations but rather capture different temporal phases of the same process.

Listening effort receives further treatment across multiple papers. Thakkar et al. find that spatial separation improves intelligibility without reducing pupil-indexed effort—and that the pupillometric measures themselves show only moderate reliability. Intelligibility and effort are not the same thing, and this dissociation has real consequences for how we evaluate interventions. Slomianka et al. study something harder to bring into the lab: triadic face-to-face interactions in noise. Listeners under these conditions shift gaze more tightly, increase saccade rates, and coordinate visual attention with greater precision. These are compensatory multimodal strategies, and they are essentially invisible in standard two-person laboratory paradigms. Kamal et al. show that increasing working memory load reduces P3a responses to novel sounds without affecting earlier change-detection components, suggesting that involuntary attention capture can be modulated top-down—though not eliminated.

Two contributions offer theoretical frameworks that situate these empirical findings in broader context. Strauss et al. develop an evolutionary cognitive neuroscience account of effortful listening, arguing that the difficulty of understanding speech in noise reflects a fundamental mismatch between an auditory system shaped by Pleistocene conditions and the acoustic environment of the Anthropocene. Their application of the free energy principle formalizes why irrelevant sounds capture attention and why that capture is metabolically costly. The practical implication that hearing technology should aim to augment rather than simply restore represents a genuinely different design goal, one with consequences for how efficacy is defined and measured. Grinfeder et al. move in a different direction, asking whether human listeners can estimate the number of biological sound sources in natural soundscapes. They can, though imprecisely, and perceived biodiversity appears to be capped at roughly three sources. This cap mirrors limits found in cocktail-party research, suggesting that the auditory grouping mechanisms shaped by evolutionary pressure to parse natural scenes are the same ones we now rely on for speech. The connection between ecological acoustics and speech perception has barely been explored.

Finally, Zhang et al. demonstrate that speech-evoked mismatch negativity can serve as an objective correlate of cochlear implant performance—a bridge between neural coding research and clinical assessment that is particularly valuable for patients who cannot complete standard behavioral testing.

## Conclusion

What do 16 papers on auditory neuroscience have in common? At one level, not much: the methods range from single-unit recordings in gerbil auditory nerve to pupillometry in human listeners to structural network analysis in NIHL patients. But a common architecture runs through all of them: from immediate effects on cue use and processing effort, to adaptive and compensatory neural responses, to the cumulative consequences of chronic noise exposure. Single measures routinely miss important parts of what is happening, and the environments in which hearing actually matters—the noisy restaurant, the pandemic classroom, the concert venue, the farm—impose demands that controlled laboratory conditions often cannot capture. By integrating behavioral, neural, physiological, and real-world evidence, these papers collectively underscore the importance of studying auditory perception within the complex and variable environments in which communication actually occurs.

